# Mild Alkalization Acutely Triggers the Warburg Effect by Enhancing Hexokinase Activity via Voltage-Dependent Anion Channel Binding

**DOI:** 10.1371/journal.pone.0159529

**Published:** 2016-08-01

**Authors:** Cung Hoa Thien Quach, Kyung-Ho Jung, Jin Hee Lee, Jin Won Park, Seung Hwan Moon, Young Seok Cho, Yearn Seong Choe, Kyung-Han Lee

**Affiliations:** 1 Department of Nuclear Medicine, Samsung Medical Center, Seoul, Korea; 2 Samsung Advanced Institute for Health Sciences & Technology, Sungkyunkwan University School of Medicine, Seoul, Korea; University of Nebraska Medical Center, UNITED STATES

## Abstract

To fully understand the glycolytic behavior of cancer cells, it is important to recognize how it is linked to pH dynamics. Here, we evaluated the acute effects of mild acidification and alkalization on cancer cell glucose uptake and glycolytic flux and investigated the role of hexokinase (HK). Cancer cells exposed to buffers with graded pH were measured for ^18^F-fluorodeoxyglucose (FDG) uptake, lactate production and HK activity. Subcellular localization of HK protein was assessed by western blots and confocal microscopy. The interior of T47D breast cancer cells was mildly alkalized to pH 7.5 by a buffer pH of 7.8, and this was accompanied by rapid increases of FDG uptake and lactate extrusion. This shift toward glycolytic flux led to the prompt recovery of a reversed pH gradient. In contrast, mild acidification rapidly reduced cellular FDG uptake and lactate production. Mild acidification decreased and mild alkalization increased mitochondrial HK translocation and enzyme activity. Cells transfected with specific siRNA against HK-1, HK-2 and voltage-dependent anion channel (VDAC)1 displayed significant attenuation of pH-induced changes in FDG uptake. Confocal microscopy showed increased co-localization of HK-1 and HK-2 with VDAC1 by alkaline treatment. In isolated mitochondria, acidic pH increased and alkaline pH decreased release of free HK-1 and HK-2 from the mitochondrial pellet into the supernatant. Furthermore, experiments using purified proteins showed that alkaline pH promoted co-immunoprecipitation of HK with VDAC protein. These findings demonstrate that mild alkalization is sufficient to acutely trigger cancer cell glycolytic flux through enhanced activity of HK by promoting its mitochondrial translocation and VDAC binding. This process might serve as a mechanism through which cancer cells trigger the Warburg effect to maintain a dysregulated pH.

## Introduction

The Warburg effect refers to the inclination of cancer cells to produce energy predominantly through a heightened rate of glycolysis and lactate production [1]. This likely represents a response to an increased demand for energy and biomass substrates to promote their survival and proliferation [2,3]. This metabolic tumor hallmark is also widely exploited in the clinics for ^18^F-fluorodeoxyglucose (FDG) positron emission tomography (PET) imaging of malignant disease [4]. Despite its pivotal role in tumor biology, however, efforts to target the Warburg effect for cancer treatment to date have been met with limited success [5].

To fully understand how cancer cells control a balance between glycolytic and oxidative metabolism, it is pertinent to recognize a link between this feature and pH dynamics. Cancer cells have a reversed pH gradient with a slightly elevated intracellular pH despite an acidic microenvironment [6], and this property has a central role in tumor biology. Accordingly, there is interest in manipulating the forces behind this dysregulated pH to regress tumor growth and progression [6]. Cellular alkalinity represents a common pathway in tumorigenicity induced by oncogenes and growth factors [7–9]. Recent studies showed that brief exposure to alkaline pH can induce cancer cell rounding with enhanced invasive potential [10], and cause formation of bleb-like structures related to cell polarity and movement [11]. Intriguingly, elevation of intracellular pH is currently proposed as an integral explanation for the Warburg effect [12–15]. Indeed, it has been suggested that tumor cell alkalosis and the Warburg effect may actually represent different aspects of the same biological phenomenon [16]. In the presence of adequate oxygen, intracellular pH plays a key role in determining the way cancer cells handle glucose. The two modes of glucose metabolism are both pH-sensitive but in opposite directions. Hence, alkaline and acidic cellular pH tends to drive energy metabolism toward glycolysis and oxidative phosphorylation, respectively [14]. Contrariwise, the Warburg effect serves a functional role in maintaining pH dysregulation by augmenting acid generation through a shift of metabolism toward glycolysis. A previous study demonstrated that control of steady state lactate production occurs through transcriptional regulation of glycolytic elements [17]. However, it has been observed that cellular acidification and alkalization stimulates shifts of metabolic patterns in a rapid manner [18–20], which cannot be explained by the delayed effects of transcriptional control.

The Warburg effect is mediated by a series of glycolytic enzymes, a key element of which is hexokinase (HK). HK is the first enzyme of the glycolytic pathway, and is frequently harnessed for tumor progression [21]. Among allosteric factors that control glycolysis, H+ is considered to have one of the most significant factors on the activity glycolytic enzymes [6]. Accordingly, the catalytic activity of HK has been shown to be dependent on surrounding pH [22,23]. However, the precise role of HK activity in maintaining pH homeostasis in living cells remains to be clarified. In cancer cells, a portion of HK is bound to an outer mitochondrial membrane protein called voltage-dependent anion channel (VDAC). This key channel protein integrates cellular energy metabolism by controlling the influx and efflux of metabolites and ions. Many cancer cells have increased expression of VDAC, which serves as an anchor point for mitochondria-interacting proteins including HKs and provides metabolic and survival benefits [24]. VDAC sequences have been identified that interact with and bind HK-1 and HK-2 [25]. VDAC binding offers HK enzymes with preferential access to mitochondrial ATP and protection from product inhibition [26–29], thereby pushing glucose metabolism toward glycolytic flux and lactate generation. Although various signal transduction stimuli have been implicated in regulating HK and VDAC interaction, the influence of cellular pH has not been explored.

In this study, we investigated the role of cellular pH and HK enzymes on the glycolytic behavior of cancer cells.

## Materials and Methods

### Cell culture and pH buffers

Human breast cancer T47D, MCF-7 and MDA-MB-468 cells and mouse colon cancer CT-26 cells were from the Korean Cell Line Bank. Breast cancer cells were maintained in RPMI 1640 medium and CT-26 cells in low-glucose DMEM medium (Lonza, Switzerland), supplemented with 10% fetal bovine serum, 100 U/ml penicillin and 100 mg/L streptomycin at 5% CO_2_ in 37°C. Experiments were performed 2 or 3 days following seeding, when cell confluence was approximately 90%.

Cells were alkalinized or acidified with pH buffers prepared from Hank's balanced salt solution (Gibco Invitrogen) containing 0.2% bovine serum albumin and 1 g/L glucose. Solutions at pH 6.2 and pH 6.8 were buffered with 10 mM MES (Sigma); solutions at pH 7.2, 7.4 and pH 7.8 were buffered with 10 mM HEPES (Sigma). Solution pH was titrated immediately before use.

### FDG uptake measurement

The culture media of cells in 12-well plates was replaced with buffers at indicated pH after brief washing with the buffer. Cells were incubated for 40 min at 37°C, 5% CO_2_ with 185 kBq of FDG. Cells were rapidly washed twice with cold phosphate buffered saline (PBS) and lysed in 0.1 N NaOH. Cell-associated radioactivity was measured on a high energy γ-counter (Wallac).

### Extracellular and intracellular pH measurement

Extracellular pH was measured directly with a pH meter using buffer aliquots from cells from 6-well plates. Intracellular pH was measured using 96-well, flat, black, transparent-bottom plates (Corning, NY) and the fluorescent pH indicator 2′,7′-bis-(2-carboxyethyl)-5-(and-6)- carboxyfluorescein acetomethyl (BCECF-AM; Molecular Probes). To calibrate fluorescent intensity ratios with intracellular pH, cells were incubated with 5 μM BCECF-AM for 30 min at 37°C, 5% CO_2_ in high potassium buffer (100 mM KCl, 50 mM 4-(2-hydroxyethyl)-1-piperazineethanesulfonic acid [HEPES], 1 mM MgCl_2_, 2 mM CaCl_2_, 5 mM NaCl) at pH 6.2 to 7.8 (adjusted with NaOH). Measurements were performed with 10 μM nigericin (Molecular Probes), a K^+^/H^+^ ionophore that equilibrates extracellular and intracellular pH in the presence of depolarizing concentrations of potassium. After removal of solutions, fluorescent intensities were quantified on a fluorescent microplate reader (Spectrafluor Plus; Tecan Group Ltd., Switzerland). The ratio of fluorescence at an emission wavelength of 535 nm from dual excitation wavelengths of 485 and 430 nm was calculated. A calibration curve confirmed a linear increase of the ratio with pH increments from 6.2 to 7.8, and a standard curve was used to measure the intracellular pH. This technique has been previously shown to provide accurate measurement of intracellular pH of living cells [30,31].

### Lactate assay

Lactic acid concentration was measured from aliquots of buffers of cells using Cobas assay kits (Roche/Hitachi, Mannheim, Germany) following the manufacturer’s instructions. Lactate was converted to pyruvate and hydrogen peroxide, which underwent enzymatic reactions that produced a dye measured by a spectrophotometer. Final lactate concentrations were expressed in mU/mg.

### Hexokinase activity assay

To obtain subcellular fractionations, cells in 150 mm plates were scraped on ice and disrupted by repeated passage through a 26G syringe. After cell debris and nuclei were eliminated by centrifugation at 1000*g* at 4°C for 10 min, an aliquot of the supernatant was used to measure total HK activity. The remaining supernatant was mixed with ice-cold isolation buffer containing 10 mM Tris-(4-morpholinepropanesulfonic acid), 1 mM ethylene glycol-bis(2-aminoethylether) tetra-acetic acid/Tris (pH 7.4), and 0.25 M sucrose, and centrifuged at 20,000g, 4°C for 10 min to obtain cytosolic (supernatant) and mitochondrial fractions (pellets dissolved in 0.1 ml isolation buffer). Protein concentrations of samples were determined by Bradford assays.

To measure HK activity, 50 μL of sample was added to 2.52 ml reaction mixture containing 39 mM triethanolamine, 216 mM D-glucose, 0.74 mM adenosine 5’-triphosphate, 7.8 mM magnesium chloride, 1.1 mM β-nicotinamide adenine dinucleotide phosphate, and 2.5 units of glucose 6-phosphate dehydrogenase. Formation of nicotinamide adenine dinucleotide phosphate at 25°C was monitored by 340 nm absorbance. One unit was defined as the amount of activity that phosphorylates 1 μmole of D-glucose per min at 25°C. Results were expressed as % activity per mg protein.

### siRNA transfection

Control siRNA (sc-37007), HK-1 siRNA (sc-39044), HK-2 siRNA (sc-35621), and VDAC1 siRNA (sc-42355) were from Santa Cruz Biotechnology (Dallas, TX). Lipofectamine RNAiMAX reagent and GIBCO Opti-MEM reduced serum medium were from Invitrogen life technologies (Carlsbad, CA). For transfection, 100 nM of siRNA was diluted in 50 μl Opti-MEM transfection medium (solution A). Solution B was prepared by adding 2 μl RNAimax tansfection reagent to 50 μl Opti-MEM transfection medium. Solutions A and B were mixed and incubated for 45 min at room temperature. Meanwhile, cells on 100 mm plates were trypsinized, washed twice with media without fetal bovine serum or antibiotics, and 1 x 10^4^ cells were transferred to eppendorf tubes. After adding the pre-incubated siRNA mixture, cells were transferred to a 24-well plate and incubated in a CO_2_ incubator at 37°C. After 5 h, medium containing 20% fetal bovine serum and 2% antibiotics was added without removing the siRNA mixture. After overnight incubation, fresh medium containing 10% fetal bovine serum and 1% antibiotics was replaced, and cells were further maintained for 48 h before uptake experiments.

### Confocal imaging for HK-1, HK-2, and VDAC localization

Cells were seeded on an 8-well chamber slide (Lab-Tek II, Nunc) and maintained for 24 h. After washing twice with cold PBS, cells were incubated with indicated pH buffers at 37°C for 50 min, and then fixed with 100% methanol for 15 min at -20°C, and washed twice with ice-cold sodium phosphate buffers of respective pH. Cells were first incubated with an antibody against VDAC (Cell Signaling; 1:200) diluted with PBS containing 0.1% Triton X-100 for 1 h at RT, washed, and incubated with an alexafluor594 anti-rabbit secondary antibody (1:500) for 30 min. Cells were then incubated with antibodies against HK-1 (Cell Signaling; 1:200) or HK-2 (Cell Signaling; 1:500) for 1 h at RT, washed, followed by an anti-rabbit FITC secondary antibody (1:200). Cells on slides were finally mounted with ProLong Gold antifade with DAPI (Invitrogen), and imaged with a confocal laser scanning microscope (Zeiss) using appropriate filters.

### Immunoblotting for HK-1, HK-2, and VDAC

For each sample, 30 μg protein was loaded onto a 12% SDS-polyacrylamide gel, separated by electrophoresis, and transferred to nitrocellulose membranes. After blocking with 5% skim milk in TBST at RT for 1 h, membranes were incubated overnight at 4°C with a rabbit polyclonal antibody against HK-1, HK-2 (1:1000), or VDAC (1:1000), followed by 1 h incubation at RT with horseradish peroxidase-conjugated anti-rabbit antibody (Cell Signaling; 1:5000). Immune reactive proteins were detected by an enhanced chemiluminescence system. Protein band intensities were measured using a GS-800 calibrated densitometer with Quantity One software (Bio-Rad Laboratories). Antibodies against β-actin (Abcam; 1:5000) and COX4 (Abcam; 1: 5000) were used as loading controls for cytosolic and mitochondrial samples, respectively.

We also used immunoblots to assess whether acidic pH directly promotes dissociation of HK-1 and HK-2 protein from mitochondria isolated from T47D cells. Briefly, cells were lysed by repeated passage through a 26-G syringe, and mitochondrial pellets were obtained through fractional centrifugation. After the pellets were incubated in a buffer of pH 6.2 or pH 7.8 for 50 min, protein remaining in the pellet and protein released into the supernatant were separated by gel electrophoresis, transferred to nitrocellulose membranes, and immune-blotted as above. Anti-COX4 antibody was used as loading control for pellet samples and to ensure exclusion of mitochondria for supernatant samples.

### Immunoblotting of membrane Glut1

Cells collected by scraping were disrupted by repeated passage through a 26-G syringe in 0.5 ml solution A (pH 7.4) containing 250 mM sucrose, 10 mM HEPES, 1 mM EDTA, 1 mM phenylmethylsulfonyl fluoride, and 1 μM aprotinin. After removal of cell debris by 14,000 rpm centrifugation at 4°C for 10 min, the supernatant was mixed 1:1 v/v with solution B containing 250 mM sucrose, 10 mM HEPES, and 1 mM MgCl_2_. Following incubation at 4°C for 1 h, membrane fractions were centrifuged at 55,000 rpm at 4°C for 1 h and dissolved in a minimal volume of water. Protein (15 μg) was separated by electrophoresis on a 10% SDS-polyacrylamide gel and transferred to nitrocellulose membranes. After blocking with 5% skim milk in TBST at RT for 1 h, membranes were incubated overnight at 4°C with antibody against human Glut-1 (DAKO; 1: 1000) followed by 1 h incubation at RT with horseradish peroxidase-conjugated anti-rabbit antibody (Cell Signaling; 1: 5000). Immune reactive proteins were detected and bands intensities were quantified as above.

### Hexokinase-VDAC binding

To assess VDAC binding, cells were lysed in RIPA buffer (150 mM NaCl, 1% Triton X-100, 0.5% sodium deoxycholate, 0.1% SDS, 50 mM Tris, pH 8.0, 5 mM NaF, 1 mM Na_3_VO_4_) supplemented with protease inhibitor cocktail (Sigma). Cell lysates (1 mL) were precleared by incubation with 100 μL of protein G agarose (Sigma) at 4°C for 10 min and blocked at RT for 1 h with 10 μM VDAC-binding membrane domain peptide (Calbiochem). The resultant lysate (500 μg) was immune precipitated at 4°C for 2 h with 2 μg rabbit polyclonal anti-VDAC1/2/3 (Santa Cruz), and immune complexes were captured by overnight incubation with 100 μL of protein G agarose at 4°C. Purified VDAC-protein G agarose bead complexes were washed with cold PBS and incubated at RT for 50 min with recombinant HK protein (Sigma) at indicated pH. Beads were washed with PBS and bound proteins used for western blots for HK-1, HK-2, and VDAC.

### Statistical analysis

Data are expressed as mean ± SD. Significance of differences between groups was analyzed by two-tailed unpaired Student *t*-tests. Correlation was analyzed by linear regression. *P* < 0.05 was considered statistically significant.

## Results

### Alterations of intracellular pH by alkalization and acidification

**Fig 1:**
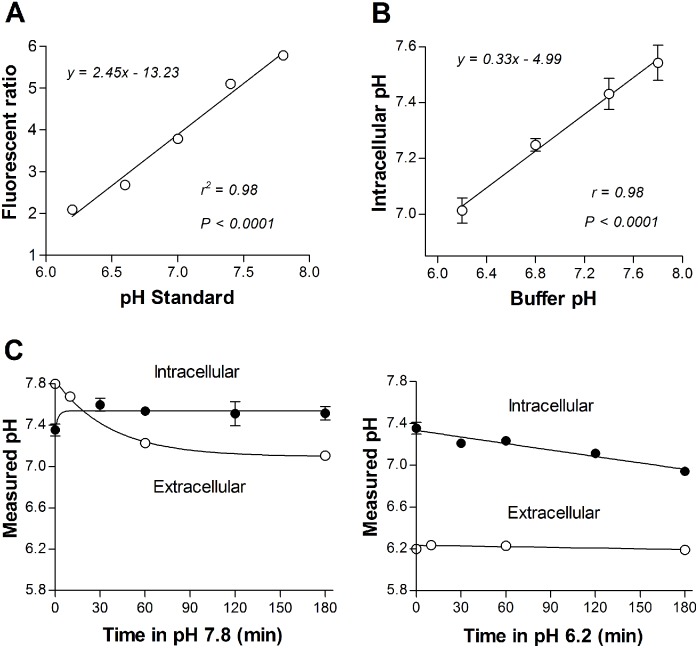
Time course of changes in cellular pH following alkaline and acidic exposure. (A) Close linear relationship between fluorescent emission ratios from BCECF-AM by excitation at 485 and 430 nm and the pH of standards applied to cell interior. (B) Linear relationship between applied buffer pH and intracellular pH of live T47D cells after 30 min. (C), Time course of extracellular pH (open circles) and intracellular pH (closed circles) over 3 h following incubation in pH 7.8 (left) or 6.2 (right). Data points are mean ± SD of triplicate samples.

Intracellular pH could be accurately measured using emitted fluorescence ratios from BCECF-AM indicators that closely correlated to pH standards applied to cell interior (Fig 1A). When this technique was applied to T47D cells exposed for 30 min to graded pH, intracellular pH was shifted toward the applied buffer pH in a close linear manner. Hence, intracellular pH shifted to 7.01 ± 0.05, 7.23 ± 0.01, 7.43 ± 0.06 and 7.54 ± 0.06 following 30 min exposure to pH 6.2, 6.8, 7.6 and 7.8, respectively (Fig 1B). Evaluation of the temporal profile showed that buffer pH 7.8 mildly alkalized T47D cell interior to a pH of 7.5 within 30 min, which then remained stable for up to 3 h. During this time, the extracellular pH exponentially decreased from 7.8 to 7.1 (Fig 1C). In comparison, buffer pH 6.2 resulted in a linear decrease of intracellular pH from 7.4 to 6.9 over 3 h, while the extracellular pH was largely unchanged (Fig 1C).

### Acidification reduces and alkalization augments cancer cell glucose uptake

**Fig 2:**
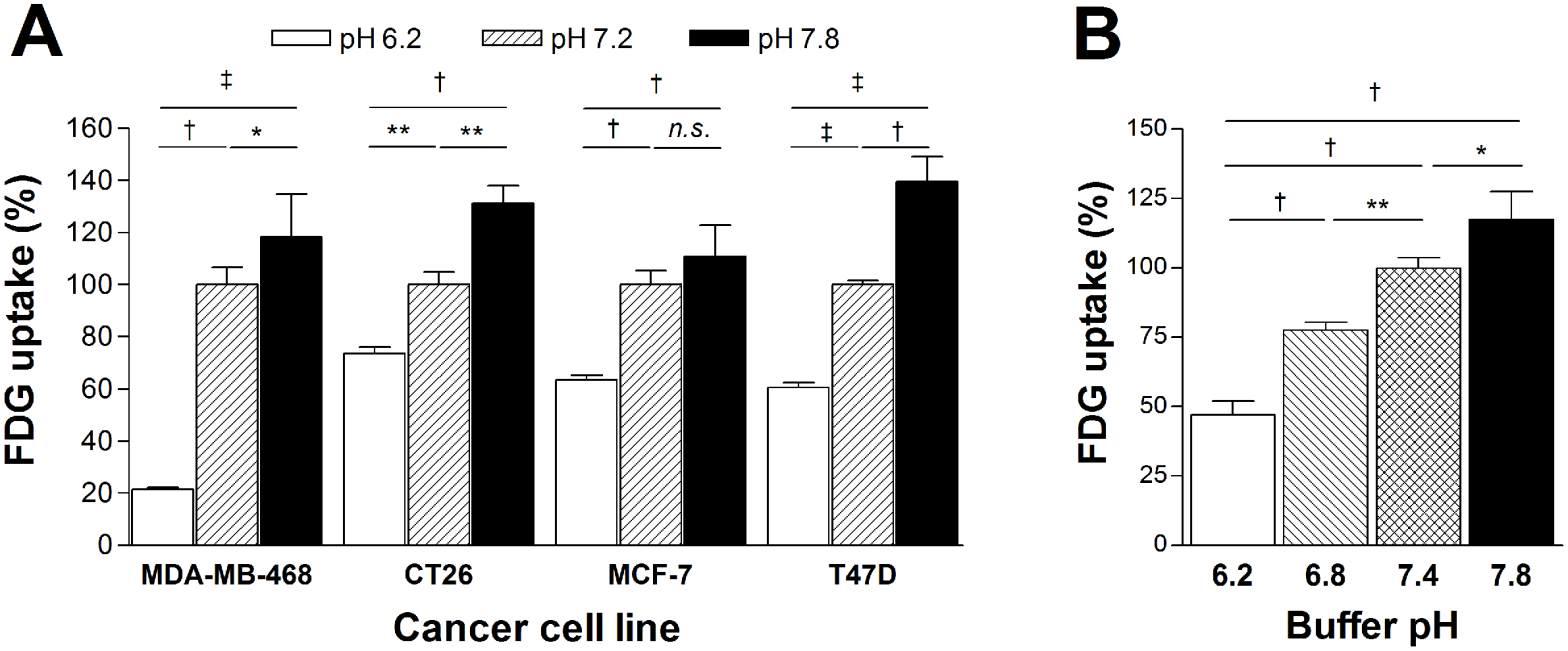
Acidification reduces and alkalization augments cancer cell glucose uptake. (A) Reduced and augmented FDG uptake in breast cancer and colon cancer cells after 10 min incubation in buffer pH 6.2 and 7.8, respectively, compared to buffer pH 7.2. (B) Linear increase of FDG uptake in T47D breast cancer cells after 10 min incubation in buffers with graded pH increments. Bars are mean ± SD of uptake (n = 3) relative to cells in pH 7.2 (A) or 7.4 (B). *, p <0.05; **, p <0.01; †, p <0.005; ‡, p <0.001; n.s., not significant.

Compared to a pH of 7.2, 10 min exposure to buffer pH 6.2 led to a rapid reduction of FDG uptake to 21.5 ± 0.9% for MDA-MB-468 cells, 73.6 ± 2.6% for CT26 cells, 63.5 ± 1.9% for MCF-7 cells, and 60.6 ± 1.8% for T47D cells (Fig 2A). Conversely, 10 min exposure to buffer pH 7.8 rapidly augmented FDG uptake to 118.4 ± 16.6% for MDA-MB-468 cells, 131.2 ± 6.8% for CT26 cells, 110.9 ± 11.8% for MCF-7 cells, and 139.4 ± 9.8% for T47D cells, compared to a pH of 7.2 (Fig 2A).

In T47D cancer cells that were used for further experiments, graded alterations of buffer pH led to a linear dose-dependent change of FDG uptake (Fig 2B). Linear regression analysis between buffer pH and relative FDG uptake showed a high correlation coefficient of 0.98 (*P* < 0.0001).

### pH change affects glycolytic flux by modulating mitochondrial hexokinase activity

**Fig 3:**
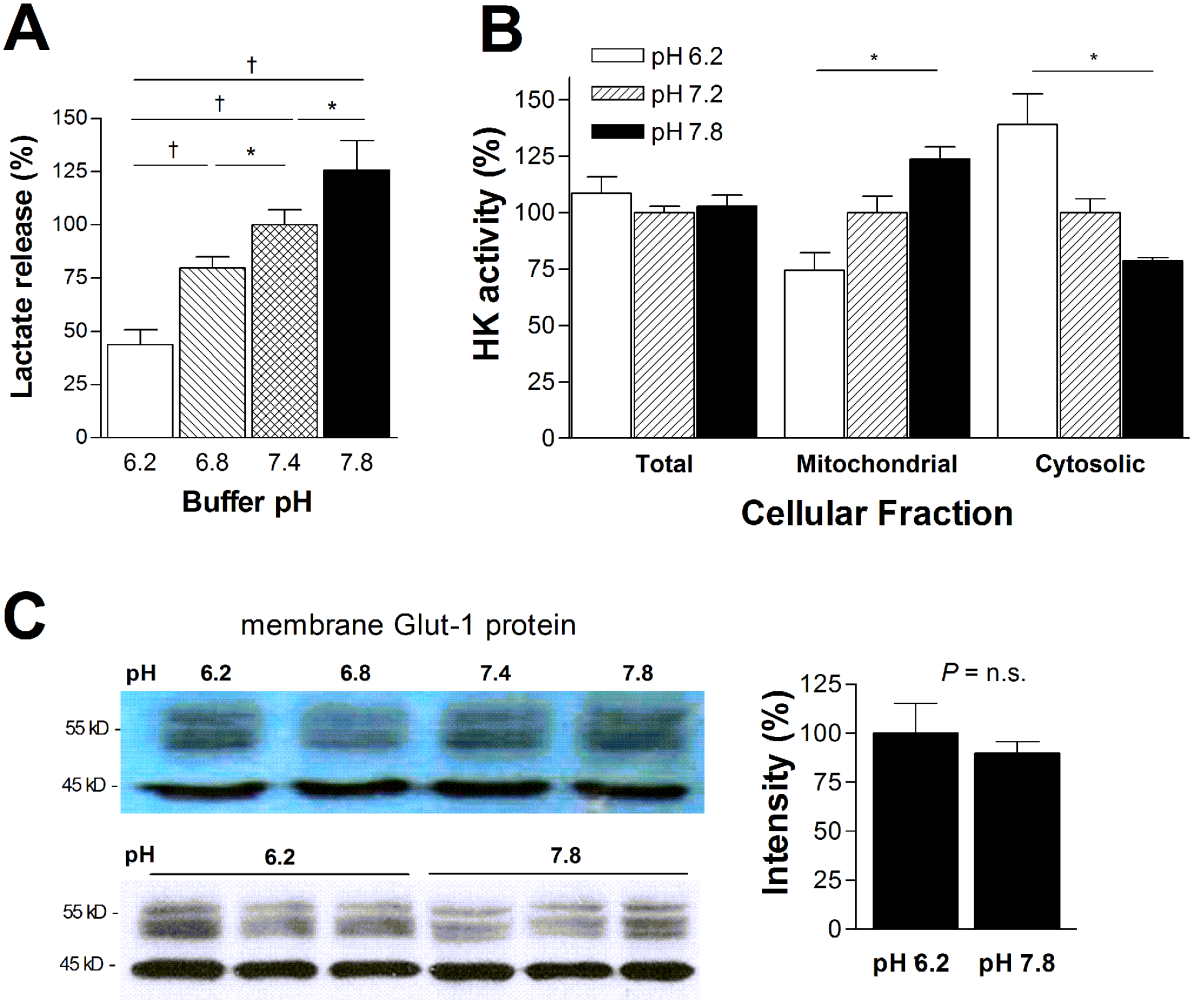
Effects of pH on glycolytic flux and hexokinase activity. (A) Linear increase of lactate production in T47D cells by exposure to graded pH increments. (B) Hexokinase activity in total cell lysates, mitochondrial fractions, and cytosolic fractions after exposure to pH 6.2, 7.2., and 7.8. (C) Membrane Glut-1 immunoblots (left) and quantified band intensities (right) after 50 min of acidic or alkaline exposure. Bars are mean ± SD (n = 3) relative to cells in pH 7.4 (A), 7.2 (B), or 6.2 (C). *, p <0.05; †, p <0.005; n.s., not significant.

Lactate release changed linearly by graded buffer pH in a manner similar to FDG uptake. Hence, buffer pH 6.2 decreased lactate production to 43.8 ± 6.7% of that at buffer pH 7.4. In contrast, buffer pH 7.8 increased lactate production to 125.8 ± 13.6%, consistent with a shift of metabolism to glycolysis (Fig 3A).

Given that glucose transporters and HK are the two major determinants of cancer cell FDG uptake, we evaluated how these components were influenced by varying pH. As a result, HK activity in the mitochondrial fraction increased to 123.8 ± 5.3% by buffer pH 7.8 and decreased to 74.5 ± 7.8% by buffer pH 6.2, compared to cells in a buffer pH of 7.2 (Fig 3B). Cytosolic HK activity displayed a reversed response to buffer pH (Fig 3B). In contrast, membrane Glut-1 expression level was unaffected by buffer pH (Fig 3C).

### Alkalization shifts cancer cell HK localization from the cytosol to mitochondria

**Fig 4:**
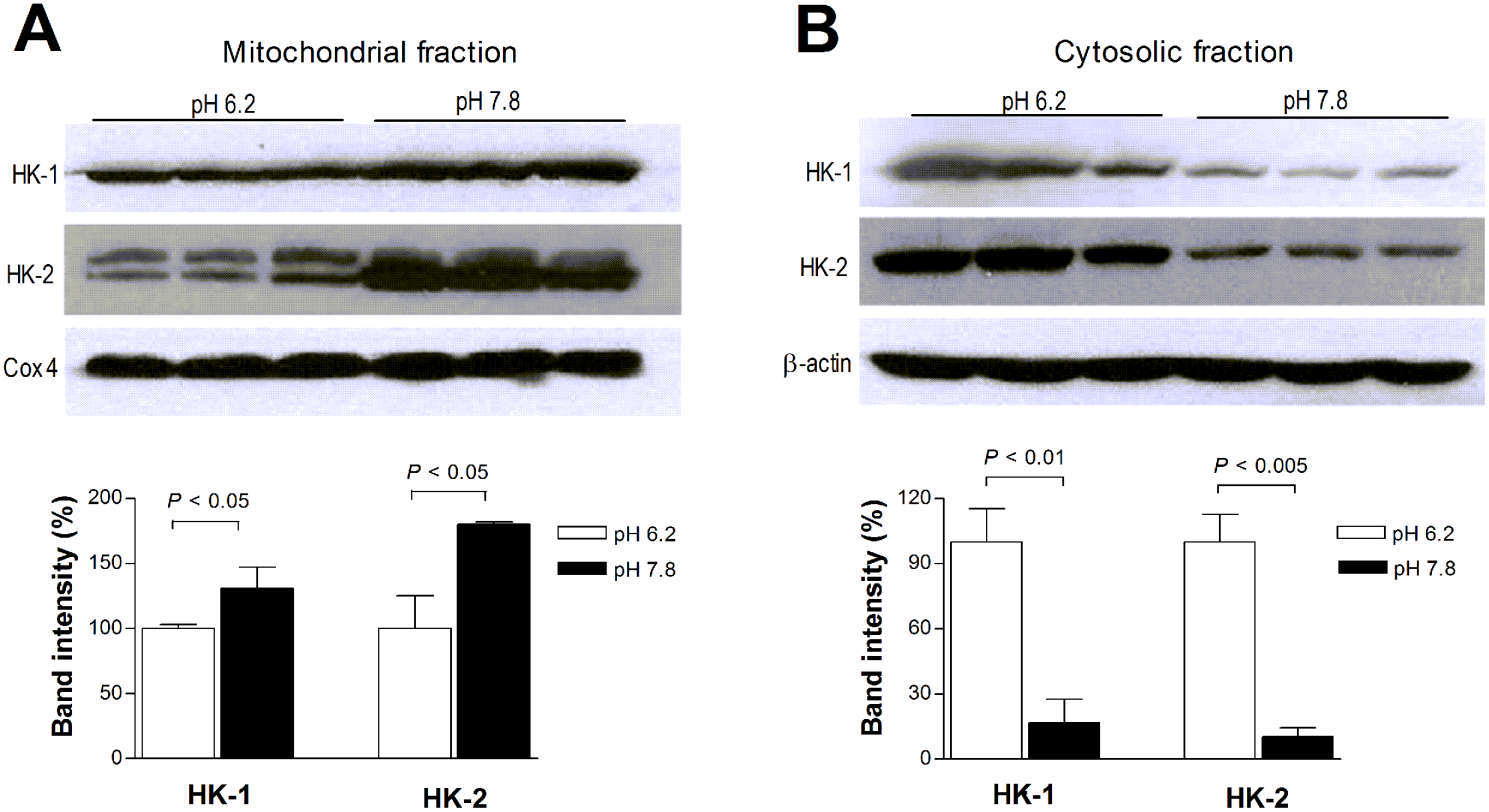
Subcellular localization of hexokinase (HK) by acidification and alkalization. (A,B) Immunoblots of HK-1 and HK-2 protein (top) and quantified band intensities (bottom) in mitochondrial (A) and cytosolic fractions (B) of T47D cells following 50 min incubation in pH 7.8 or 6.2. HK band intensities of mitochondrial and cytosolic fractions were normalized for loading using COX4 and β-actin bands, respectively. Bars are mean ± SD of band intensities (n = 3) relative to cells in pH 6.2.

To assess how alkalization influences the subcellular localization of HK, T47D cells were incubated in alkali or acidic buffers for 50 min, and subcellular fractions were analyzed by Western blotting. Buffer pH 7.8 caused significant 1.3 ± 0.2 and 1.8 ± 0.0 fold increases in the mitochondrial fraction of HK-1 and HK-2 protein, respectively, compared to buffer pH 6.2 (Fig 4A). This was accompanied by substantial reductions in the cytosolic fraction of HK-1 and HK-2 protein to 0.2 ± 0.1 and 0.1 ± 0.0-fold, respectively, of that in buffer pH 6.2 (Fig 4B).

### HK-1, HK2 and VDAC1 siRNA attenuates the effect of pH on FDG uptake

**Fig 5:**
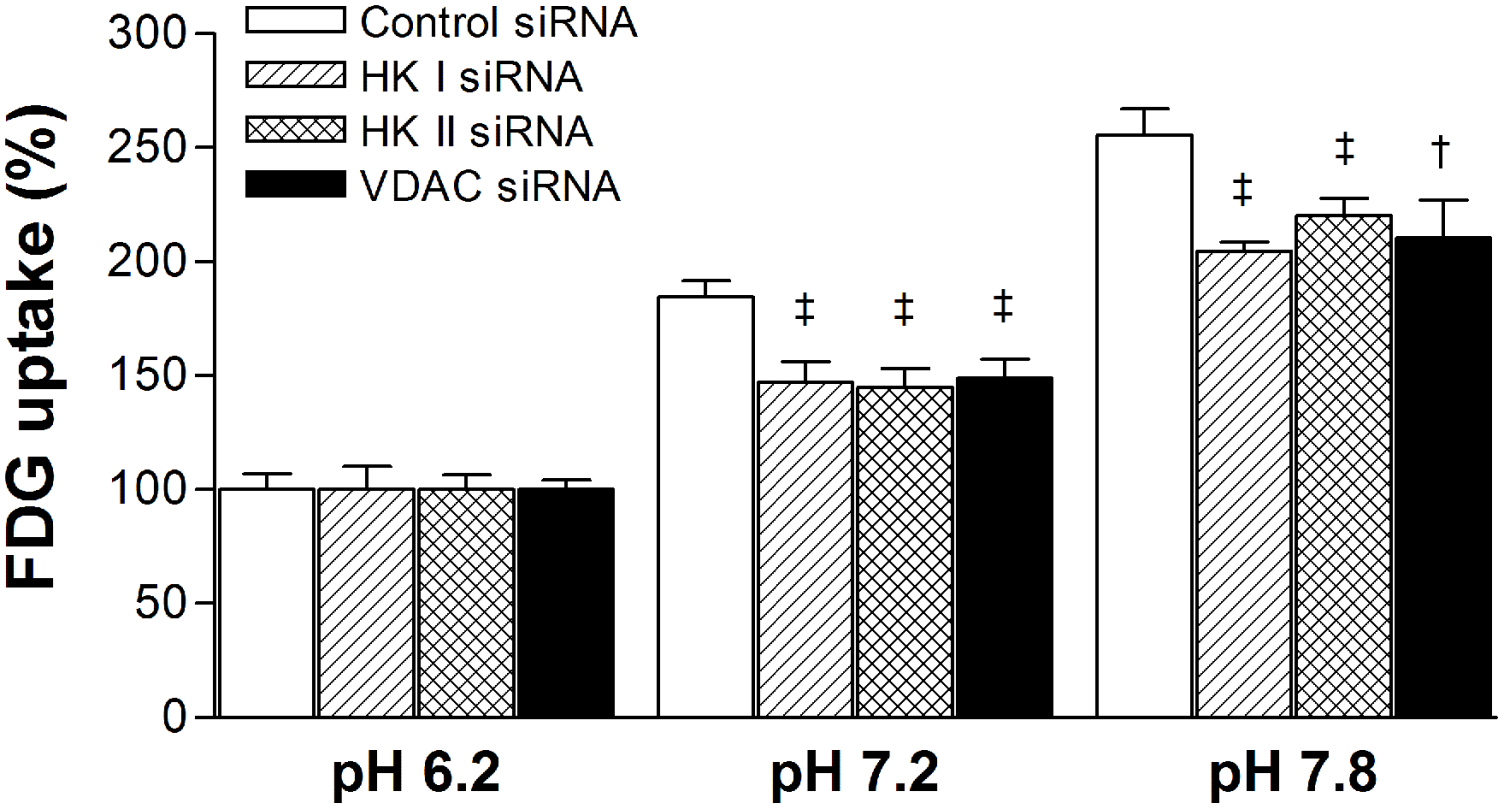
Effects of HK-1, HK-2 and VDAC1 siRNA on pH-modulated glucose uptake. Relative FDG uptake of T47D cells after transfection with specific siRNA against HK-1, HK-2, or VDAC1. Bars are mean ± SD of six samples from two separate experiments. †, p <0.0005; ‡, p <0.0001, compared to cells transfected with respective siRNA and incubated in pH 6.2.

When cells were transfected with specific siRNA against HK-1, HK2, or VDAC1, the ability of buffer pH to influence FDG uptake was significantly attenuated. Hence, whereas FDG uptake in cells transfected with control siRNA was increased to 184.6 ± 7.0% by pH 7.2 compared to pH 6.2, this was attenuated to 147.2 ± 8.8%, 144.9 ± 8.3% and 148.8 ± 8.4% in cells transfected with siRNA against HK-1, HK2, and VDAC1, respectively (Fig 5). Similarly, FDG uptake that was increased to 255.7 ± 11.3% by pH 7.8 (compared to pH 6.2) in cells transfected with control siRNA, was attenuated to 204.5 ± 4.2%, 220.4 ± 7.5% and 210.4 ± 16.8% in cells transfected with respective specific siRNA (Fig 5).

### Effects of pH on colocalization of HK-1 and HK-2 with VDAC

**Fig 6:**
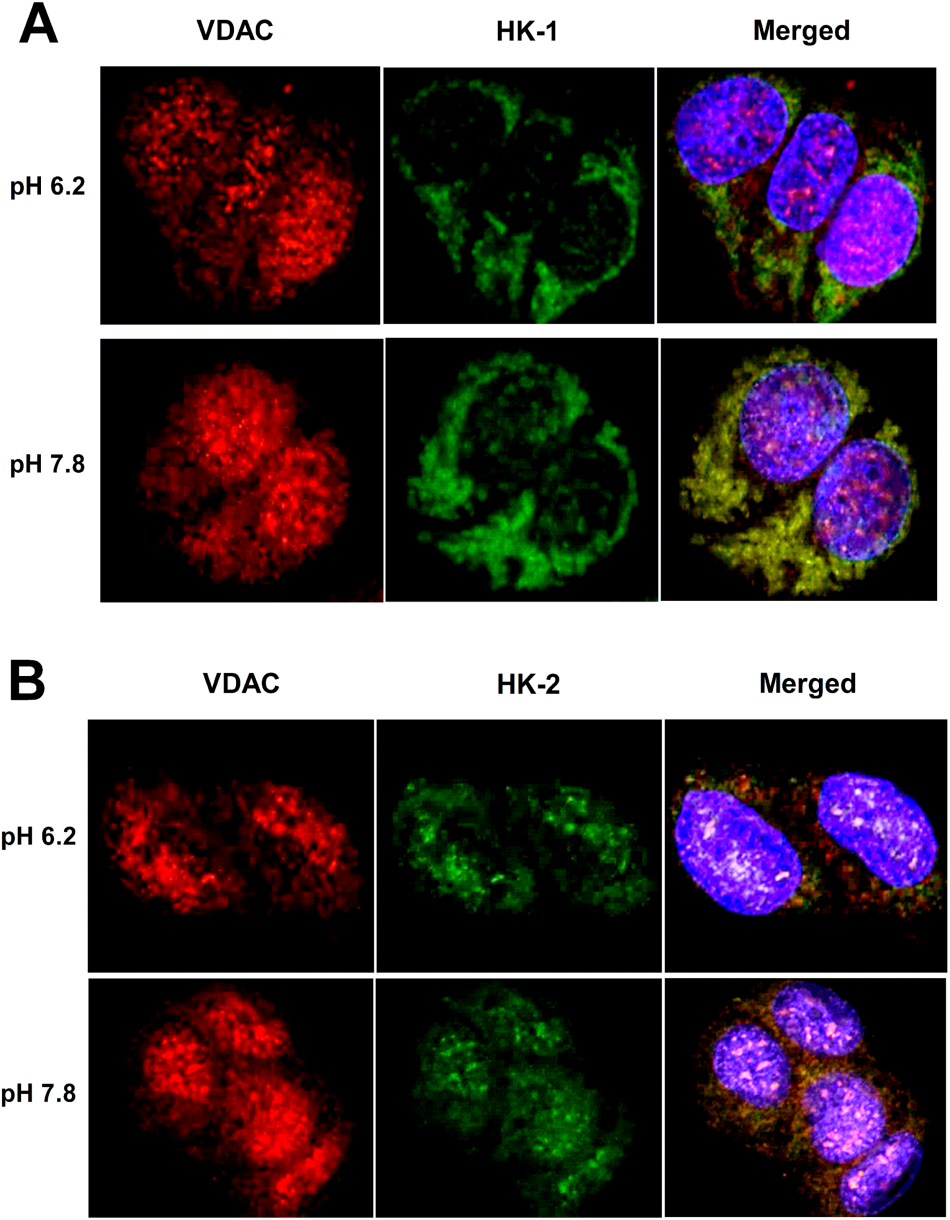
Effects of pH on co-localization of HK-1 and HK-2 with VDAC. (A,B) Confocal microscopy images of T47D cells double stained for VDAC and HK-1 (A) or VDAC and HK-2 (B) following incubation at pH 6.2 or 7.8.

Confocal microscopy images of T47D cells co-stained for VDAC and HK-1 or VDAC and HK-2 demonstrated increased co-localization of HK-1 and HK-2 with VDAC in cells incubated at pH 7.8 compared to pH 6.2 (Fig 6).

### Acidic pH promotes HK protein dissociation from mitochondrial pellets

**Fig 7:**
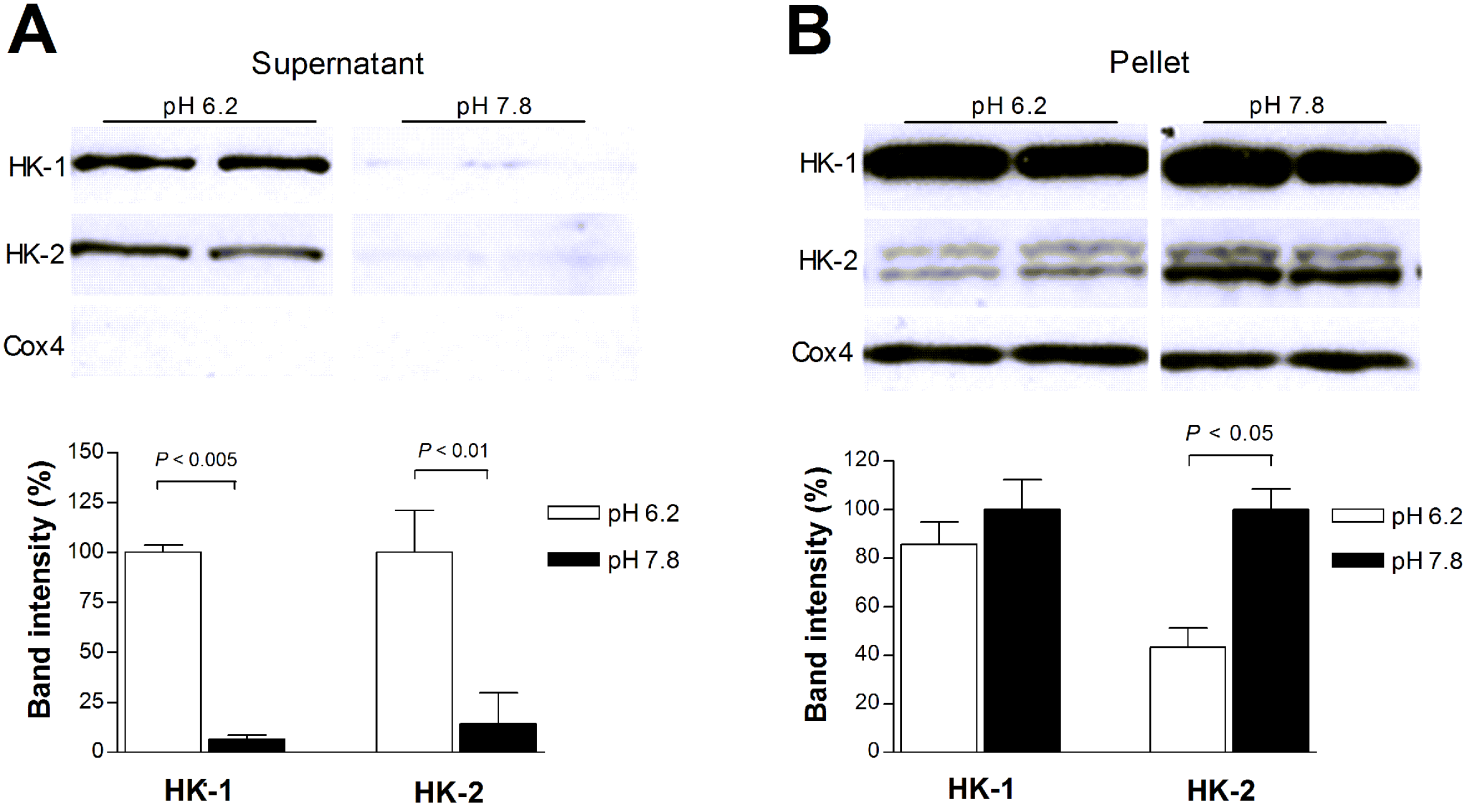
Effect of pH on HK association with mitochondria. (A) Immunoblots (top) and quantified band intensities (bottom) of free HK-1 and HK-2 protein dissociated from isolated mitochondria into the supernatant following exposure to pH 6.2 or 7.8. (B) Immunoblots and quantified band intensities of HK-1 and HK-2 protein remaining in the mitochondrial pellets following pH exposure as above. Band intensities of HK from the mitochondrial pellet were normalized by COX4 bands. In supernatants, negative COX-4 bands confirmed the absence of mitochondria in the samples. Bars are mean ± SD of band intensities (n = 2) relative to that at pH 6.2 (A) or pH 7.8 (B).

When mitochondria isolated from T47D cells were incubated for 50 min in buffer pH 6.2, there were large amounts of free HK-1 and HK-2 protein released into the supernatant, consistent with increased dissociation from mitochondria (Fig 7A). In contrast, mitochondria incubated in buffer pH 7.8 showed little free HK-1 and HK-2 in the supernatant, which was only 6.6 ± 2.1% and 14.4 ± 15.5%, respectively, of that at pH 6.2 (Fig 7A). This was accompanied by increased amounts of HK-1 (116.8 ± 10.8%) and HK-2 protein (230.3 ± 19.9%) remaining in the mitochondrial pellet at pH 7.8 compared to pH 6.2 (Fig 7B).

### Alkaline pH promotes binding of purified VDAC and HK protein

**Fig 8:**
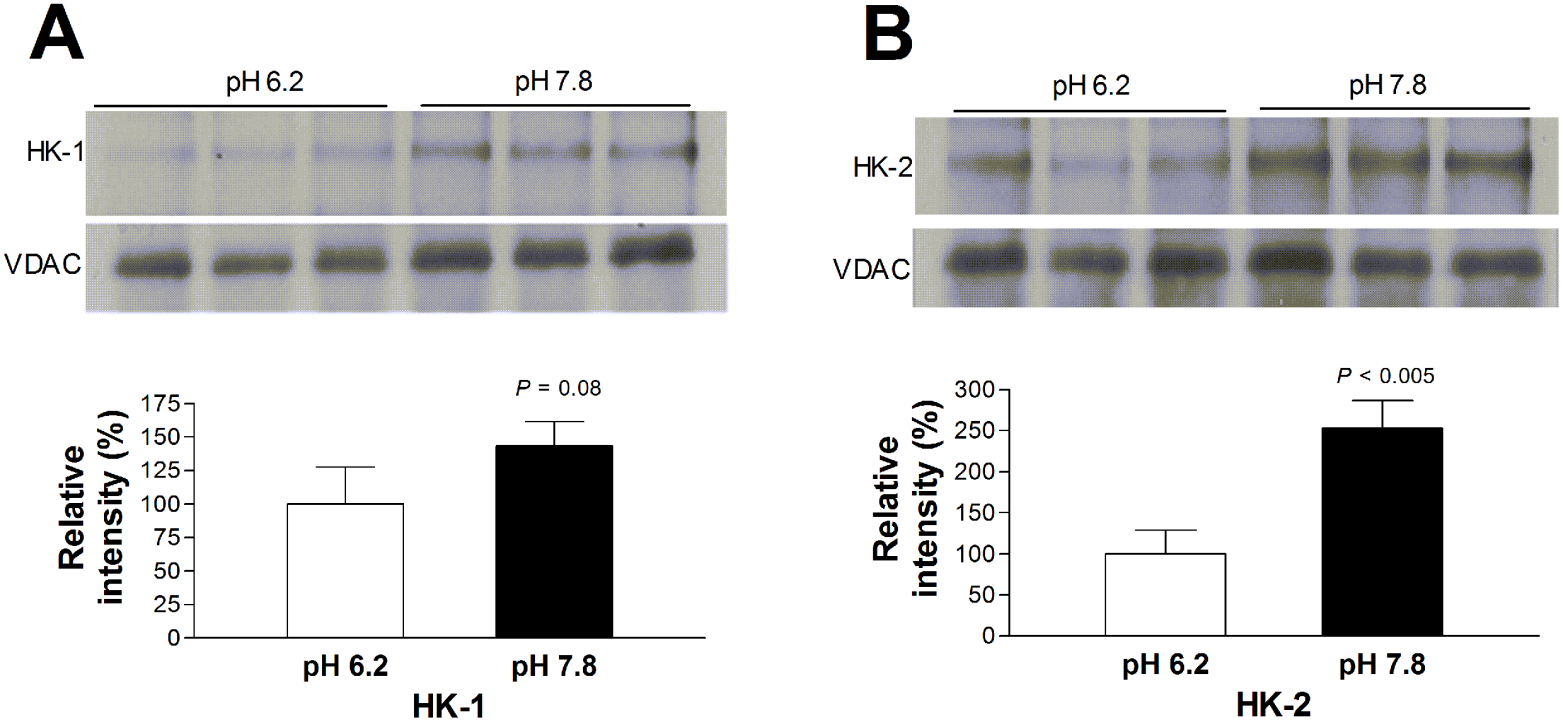
Effect of pH on HK-VDAC binding. (A, B) Immunoblots (top) and quantified band intensities (bottom) of recombinant HK-1 (A) and HK-2 (B) co-immunoprecipitated with purified VDAC protein. Recombinant HK and purified VDAC protein were pre-incubated in pH 6.2 or 7.8 for 50 min. Bars are mean ± SD (n = 3) of band intensities normalized by VDAC bands relative to that at pH 6.2.

Finally, when we incubated purified VDAC protein with recombinant HK enzymes, significantly greater amounts of HK-2 co-precipitated with VDAC protein following incubation in buffer pH 7.8 (213.0 ± 28.2% of that at pH 6.2). HK-1 also showed a tendency of greater co-precipitation with VDAC by alkaline pH (143.1 ± 18.1%), although the difference did not reach statistical significance (Fig 8A & 8B).

## Discussion

In this study, cancer cells were shown to acutely respond to mild acidification and alkalization by rapidly decreasing and increasing glucose uptake, respectively. Increased glucose uptake by mild alkalization was accompanied by enhanced glycolytic flux and lactate extrusion, which promoted pericellular acidification and recovery of a reversed pH gradient.

Although the extracellular pH of tumors is generally reported in the range of 6.5 to 6.9, a previous study in mice demonstrated that simply adding bicarbonate to drinking water could raise extracellular tumor pH from 7.0 to 7.4 [32]. This measurement was based on chemical shifts of exogenous 3-aminopropylposphonate using volume-selective ^31^P magnetic resonance spectra. In our study, exposure to mild alkaline pH caused the cytosolic pH of live T47D cells to shift from a baseline of 7.36, toward the direction of pericellular pH within 30 min. Hence, there was mild but brisk intracellular alkalization when cells were incubated in buffers with elevated pH. Alkalization has been observed to stimulate glucose uptake in various cell types [18–21]. In our study with T47D breast cancer cells, the metabolic effect occurred as early as 30 min, which cannot be explained by regulation at the transcription level after 24 h as previously observed [17]. Among cellular processes, transporter activity, protein-protein interaction, and enzyme activity are directly influenced by ambient pH. Previous studies suggested that increased transport may be responsible for the elevated glucose uptake by alkaline pH in rat liver and Ehrlich ascites-tumor cells [19,20]. Intracellular transport of glucose in most cancers including T47D cells occurs predominantly through Glut-1 [33]. However, we observed no change in membrane Glut1 expression by cellular alkalization or acidification. A recent study suggested that increased glucose uptake by pH elevation may be associated with increased activity rather than expression of Glut-1 [34]. Thus, although this possibility cannot be excluded, our experiments show that membrane Glut-1 content is not influenced by cellular pH.

HK is a key element of the Warburg effect that regulates glucose flux between mitochondrial respiration and glycolytic metabolism. In vitro kinetic experiments with purified HK enzyme have shown that the rate of glucose phosphorylation is low under acidic conditions [22], whereas it increases under alkaline conditions [23]. However, whether HK activity inside living cancer cells is influenced by cellular pH has remained unclear. Our results demonstrate that alkalized T47D cells show a 2-fold increase in mitochondrial HK activity. Given the minor elevation of intracellular pH that was observed, this level of increase in HK activity is substantially greater than expected from direct kinetic effects alone. Furthermore, HK activity in the cytosolic fraction was actually reduced by alkalization. Together, these findings indicate that major enhancement of HK activity by mild cellular alkalization occurs through mitochondrial translocation of the enzyme. This was confirmed by increased recovery of HK-1 and HK-2 from mitochondrial fractions of alkalized cells, accompanied by decreased recovery from cytosolic fractions. A previous study permeabilized glioma cells with ionophores to allow direct contact of cytosolic proteins with buffer pH and observed changes in subcellular HK distribution [35]. Our results using intact cancer cells demonstrate that induction of minor changes in intracellular pH is sufficient to change the subcellular distribution and activity of HK enzymes. Interestingly, we noticed that HK-2 appeared as two bands for mitochondrial samples whereas it appeared as a single band in cytosolic samples. A previous study on cancer cells with a single HK mRNA species reported that two types of HK protein with molecular masses of 115 and 107 KDa were present in the mitochondria [36]. As was the interpretation in that study, the dual band observed in our study may also be the result of post-translational modification of HK-2 protein in the mitochondria. However, this possibility will require further confirmation.

A key finding of our study was that pH conditions modulated HK-1 and HK-2 binding to VDAC protein. Our experiments showed that the ability of pH to modulate glucose uptake was significantly attenuated when cancer cells were transfected with specific siRNA against HK-1, HK-2 or VDAC. This indicates that all of these proteins are at least partly involved in the metabolic effect of pH change. Furthermore, confocal microscopy results support that HK-1 and HK-2 co-localization with VDAC is increased when cells are exposed to alkaline pH. HK strategically bound to VDAC have preferential access to mitochondria-generated ATP and are protected from product inhibition [26–29]. HK-VDAC binding thus helps cancer cells force glucose flux through the glycolytic pathway, promoting tumor proliferation and extracellular acidification. HK-1 has N-terminal residues that interact with part of VDAC1 [37]. Titratable groups in HK proteins that have pH-dependent protonation states may thus contribute to increased VDAC binding under alkaline conditions.

In conclusion, mild alkalization of cancer cells is sufficient to acutely enhance glycolytic flux and lactate generation. This metabolic response is mediated by enhanced HK activity via mitochondrial translocation and VDAC binding. Thus, augmented HK-VDAC binding might be a primary mechanism through which cancer cell alkalization triggers the Warburg effect and maintains a dysregulated pH.
